# Rolling circle reverse transcription enables high fidelity nanopore sequencing of small RNA

**DOI:** 10.1371/journal.pone.0275471

**Published:** 2022-10-10

**Authors:** Sean Maguire, Shengxi Guan

**Affiliations:** New England Biolabs, Inc., Beverly, MA, United States of America; Sichuan University, CHINA

## Abstract

Small RNAs (sRNAs) are an important group of non-coding RNAs that have great potential as diagnostic and prognostic biomarkers for treatment of a wide variety of diseases. The portability and affordability of nanopore sequencing technology makes it ideal for point of care and low resource settings. Currently sRNAs can’t be reliably sequenced on the nanopore platform due to the short size of sRNAs and high error rate of the nanopore sequencer. Here, we developed a highly efficient nanopore-based sequencing strategy for sRNAs (SR-Cat-Seq) in which sRNAs are ligated to an adapter, circularized, and undergo rolling circle reverse transcription to generate concatemeric cDNA. After sequencing, the resulting tandem repeat sequences within the individual cDNA can be aligned to generate highly accurate consensus sequences. We compared our sequencing strategy with other sRNA sequencing methods on a short-read sequencing platform and demonstrated that SR-Cat-Seq can obtain low bias and highly accurate sRNA transcriptomes. Therefore, our method could enable nanopore sequencing for sRNA-based diagnostics and other applications.

## Introduction

Small RNAs (sRNAs) are a diverse class of non-coding RNA that include microRNA (miRNA), small nucleolar RNA (snoRNA), tRNA fragments (tRNAf) and piwi-interacting RNA (piRNA) among others. They are important in a variety of cellular functions, including development and regulation of gene expression [1]. Furthermore, they covary with conditions such as cancer [2–4], infectious disease [5] and cardiovascular disease [6]. miRNAs have tissue specific expression patterns and are stable in body fluids, making them attractive biomarker candidates in minimally invasive liquid biopsies [7, 8]. Liquid biopsies involve sampling circulating DNA and RNA released from tumors or other tissues into circulation and offer many advantages over traditional biopsies, including non-invasive screening and early detection as well as the ability to monitor disease progression to guide real time treatment decisions [9]. Current methods for detecting sRNAs require extensive library preparations, expensive infrastructure to sequence and suffer from biased sequence representation [10–17].

Nanopore sequencers, in particular, the commercially available sequencers from Oxford Nanopore Technologies (ONT), offer many advantages for diagnostic applications, including low costs for equipment and consumables. Unlike other sequencing technologies, where data are analyzed after collection, nanopore devices are capable of real time analysis and on-chip target enrichment [18], making them attractive for point of care and resource limited environments. While these advantages have been applied for the detection of DNA and coding RNA, it is not currently possible to sequence sRNAs on a nanopore device. The relatively slow sampling rate of the nanopore coupled with the fast translocation of nucleic acids, means that molecules under approximately 200 nucleotides can’t be reliably sequenced [19]. Furthermore, the relatively high error rate precludes nanopore technology from being used for miRNA sequencing, which due to their short size (18–22 nucleotides) require high fidelity sequencing to be mapped and analyzed.

Previous approaches to detect sRNAs on ONT devices involve using chemical modifications to add bulky residues to slow the voltage driven translocation of molecules through the pore and increase the signal, however it is not possible to achieve whole transcriptome sequencing using this method [19]. In another approach, it has been shown that it is possible to sequence small DNA molecules using circularization of padlock probes followed by rolling circle DNA amplification [20], however this method required a specific probe for each target and has not been demonstrated on RNA targets. Rolling circle amplification allows for high fidelity sequencing because the original molecule is copied multiple times in tandem repeats and these repeats can be used to assemble a high accuracy consensus sequence from noisy data. Rolling circle amplification has also been used as a strategy to increase the accuracy of nanopore sequencing of long RNA, using reverse transcription followed by circularization of the cDNA product [21].

All previous approaches using rolling circle amplification to aide in nanopore sequencing have used phi29 DNA polymerase due to its high processivity and strong strand displacement. We reasoned that a highly strand displacing reverse transcriptase (RT) could achieve the same goal directly on RNA templates and would therefore allow for direct amplification of sRNA. One candidate group of enzymes to perform such a function are Group II intron reverse transcriptases. Group II introns are retrotransposons that encode a self-splicing ribozyme as well as a reverse transcriptase. To complete the retrotransposition cycle, the intron-encoded reverse transcriptase must accurately copy its cognate ribozyme which is large and highly structured. While homologous to the retroviral encoded reverse transcriptases typically used in biotechnology, this selection pressure has led to the evolution of high processivity, high fidelity and strong strand displacement activity in the Group II intron RTs [22].

In this paper we show that it is possible to perform rolling circle reverse transcription using Group II intron RTs, with much better performance than retroviral RTs. We developed an efficient sequencing strategy that allows for adapter ligation, circularization and rolling circle reverse transcription of cellular sRNAs without intermediate purifications. The resulting cDNA products can be used directly or further amplified before being sequenced on the ONT MinION device. Finally, we show that our method for converting sRNAs into concatemeric cDNAs (SR-Cat-Seq) can be used to reconstruct high-fidelity and low-bias sRNA transcriptomes from error-prone nanopore data.

## Material and methods

### Circular RNA preparation and reverse transcription

Circular RNA was prepared using 10 replicates of 20 μl reactions each containing 50 pmol of a 42nt RNA oligo (S1 Table, oligo 1), 1x T4 RNA ligase 1 buffer, 0.5 mM ATP and 30 units of T4 RNA Ligase 1 (NEB M0204L). The reactions were incubated at 25°C for 1 hour. To remove any remaining linear RNA, 2 units of XRN-1 (NEB M0338) and 500 ng of RNase R (purified in house) were directly added to the reactions and incubated for 30 minutes at 37°C. 5 replicates were combined to form 2 pooled samples and purified using the Monarch PCR & DNA Cleanup Kit (NEB T1030). These reactions were run on a 15% TBE-Urea polyacrylamide gel (Thermofisher, Waltham MA). The circular RNA species were cut from the gel and further purified using the ZR Small-RNA PAGE recovery kit (Zymo Research, Irvine CA).

The following reverse transcriptases (RTs) were tested: Avian Myeloblastosis Virus RT (AMV; NEB M0277), Moloney Murine Leukemia Virus RT (MMLV; NEB M0253), ProtoScript II RT (PSII; NEB M0368), SuperScript IV RT (SSIV; Thermofisher; Waltham, MA), WarmStart RTx (RTx; NEB M0380), Thermostable Group II Intron RT (TGIRT; InGex; Austin, Texas) and Induro RT (NEB M0681). Each reverse transcription reaction was performed under the manufacture’s recommended buffer conditions with 1 μl of the stock concentration of the enzyme. Each reaction contained 1 pmol of either circular or linear RNA oligo (S1 Table, oligo 1) and 2 pmol of DNA primer (S1 Table, oligo 2). Reactions were incubated at 38°C for 5 min to allow for primer binding and then incubated at the optimal temperature for each enzyme: 42°C (AMV, MMLV, PSII), 55°C (RTx and SSIV), or 60°C (TGIRT and Induro). Retroviral RTs (AMV, MMLV, PSII, RTx and SSIV) were allowed to react for 1 hour, while the Group II intron RT reactions were stopped by cooling down to 4°C after 15 minutes. All reactions were then purified using the Monarch PCR & DNA Cleanup Kit (NEB T1030), except the Group II intron RT reaction products (TGIRT and Induro) with the circular RNA template, which were too long to elute efficiently from silica-based columns. In these cases, NEBNext sample purification beads (E7104) were used to purify these reactions products. Each purified reaction product was then treated with 5 units of RNase H (NEB M0297) and 50 units of RNase If (NEB M0243) to remove the RNA template. Half of each reaction was run on a 1% agarose gel and the other half was run on a 15% TBE-Urea polyacrylamide gel (Thermofisher; Waltham, MA). All gels were stained with SYBR Gold Nucleic Acid Gel Stain (Thermofisher; Waltham, MA) and visualized with a Typhoon gel scanner (GE; Boston, MA).

### Preparation of splint adapter

The top RNA strand of the adapter (S1 Table, oligo 3) was adenylated using the 5’ DNA Adenylation kit (NEB E2610) and purified with Monarch RNA Cleanup Kit (NEB T2030). 10 μM top RNA strand of the adapter was mixed with 10.5 μM bottom DNA splint oligo (S1 Table, oligo 4) in the annealing buffer (10 mM Tris–HCl pH 7.5, 50 mM NaCl, 0.1 mM EDTA). The top RNA strand and the bottom DNA splint strand were annealed by heating the mixture to 82°C for 2 minutes and then cooling slowly to 4°C at a rate of 0.1°C/ second.

### SR-Cat-Seq workflow

For RNA oligos or the miRXplore synthetic reference miRNAs, 5 pmol of RNA was used as input. For human brain RNA, sRNAs (< 200 base pairs) were isolated from 10 μg of total brain RNA using RNA XP beads (Beckman Coulter) following the manufacturer’s protocol. 50 ng of isolated sRNA was used as input. For the SR-Cat-seq workflow, the RNA was first denatured by heating to 70°C for 2 minutes and placed on ice immediately. The ligation mixture was then added to the RNA. The ligation mixture contained 1x T4 RNA ligase buffer (NEB M0204), 20% PEG-8000, 0.05% Tween-20, 20 pmol of the annealed splintadapter (see above) and 200 units of T4 RNA Ligase 2 Truncated K/Q (NEB M0373) in a total volume of 20 μl. The ligation reaction was incubated at 25°C for 1 hour. Subsequently, 2 units of USER enzyme (NEB M5505) and 4 units of DNase I (NEB M0303) were added to the reaction mixture and incubated at 37°C for 30 minutes to remove the bottom DNA splint strand of the adapter. After the degradation of DNA strand, only RNA is subject to the following circularization step. The T4 RNA Ligase 2 Truncated K/Q was then heat inactivated at 75°C for 5 minutes and then cooled down to 4°C. The reaction mixture was then diluted to a total volume of 40 μL with 1x T4 RNA ligase buffer, 1 mM ATP, 10 units of T4 Polynucleotide Kinase (NEB M0201) and 30 units of T4 RNA ligase 1 (NEB M0437M). The circularization reaction was then allowed to proceed for 1 hr at 25°C. Linear RNAs were then degraded by adding the following mixture of enzymes 2uL XRN-1 (NEB M0338), 1uL 5’deadenylase (NEB M0331), RNase R (purified in house), ATP and Poly(A) Polymerase (NEB M0276L). The reaction mixture was then diluted to 80 μL with 2x Induro RT buffer (1x final concentration), 1 μM primer (S1 Table, oligo 2), 1 mM dNTPs and 100 ng of Induro RT (NEB M0681). The reaction was incubated for 38°C for 5 minutes, 60°C for 30 minutes and then 95°C for 5 minutes. The resulting cDNA was purified using 96 μl of NEBNext sample purification beads, following the manufacturer’s directions, with a modified elution protocol. The elution was incubated at 37°C for 10 min with occasional vortexing.

### Amplification-free workflow

Second strand synthesis was performed using Taq DNA polymerase. A primer (S1 Table, oligo 5) was annealed to the adapter sequence and the 5’-3’ exonuclease activity of the polymerase could remove primers annealed to the internal sequence of cDNA. The reaction mixture contained 1 μg of the rolling circle cDNA product, 1x Thermopol buffer (NEB B9004S), 1 mM dNTPs, 10 pmol primer and 5 units of Taq DNA polymerase (NEB M0273) in a total volume of 50 μL. The reaction was incubated for 95°C for 30 seconds, 62°C for 1 minute and 65°C for 20 minutes. These reactions were purified using 25 μl of NEBNext sample purification beads (E7104).

### Amplification workflow

Amplification was performed using multiple strand displacement amplification with forward and reverse primers designed to anneal to non-overlapping portions of the adapter sequence (S1 Table, oligos 6 and 7). The reactions contained approximately 40 ng of the rolling circle cDNA product, 2 μM concentration of each of the forward and reverse primers, 1 mM dNTPs, 40 μg BSA and 10 units of phi29 DNA polymerase (NEB M0269) in 1x phi29 buffer with a reaction volume of 40 μl. Reactions were incubated for 1 hour at 30°C and inactivated by heating to 65°C for 20 minutes. Reactions were diluted to 50 μL with nuclease free water and purified using 25μL of NEBNext sample purification beads (E7104). After washing the beads according to the manufacturer’s directions, beads were resuspended in 48 μL of 1x NEBuffer 2. Reactions were debranched by adding 20 units of T7 endonuclease I (NEB M0302) directly to the bead slurry and incubating the mixture for 30 minutes at 37°C. Following the debranching reaction, the supernatant was removed and purified using a fresh addition of 20 μl of NEBNext sample purification beads following the manufacturer’s directions.

### Previously published data

Short read sequencing data were previously published [16] and available at the Sequencing Read Archive (https://www.ncbi.nlm.nih.gov/sra) under project accession number: PRJNA603337. qPCR data was previously published and available in supplementary materials [16].

### Capillary electrophoresis

Adapter ligation and circularization reactions were carried out as described above using a 21 bp randomized RNA substrate with an internal FAM label (S1 Table, oligo 8). 1 μl samples were removed from the reactions and replaced with 1x buffer after the adapter ligation step, the circularization step and the RNase digestion step to assess product formation at each stage of the reaction. All reactions were carried out in quadruplicate and included controls where components were left out of the reactions, which allowed for peaks to be positively identified by comparison. Controls included no adapter, no T4 RNA Ligase 2 truncated K/Q, no T4 RNA ligase 1 and no T4 polynucleotide kinase. Peaks were quantified using Peak Scanner (Thermofisher, Waltham MA).

### Generation of consensus sequences

Reads were filtered by length (> 1000 bp) and average quality (> = 7) and then converted to FASTA format. SPADE [23] was used to detect periodic repeats in the reads and to extract consensus sequences. Iterative testing was performed to find optimal parameter tuning and the final parameters were used as follows: K-mer size = 5, sliding window size = 1000, peak height threshold = 10, gap threshold = 200, margin = 200, letter consistency threshold = 0.5. All other parameters were used with their defaults. Custom R scripts (R Core Team, version 3.6.3. https://www.R-project.org/) were used to parse the resulting genbank files from the SPADE output to collect the consensus sequences. Consensus sequences generated in this manner could have any random circular orientation, therefore, to phase them we generated all possible rotations of each consensus sequence and aligned the adapter to them using pairwise alignments with the Needleman-Wunsch algorithm as implemented in the R package Biostrings (R package, version 2.62.0. https://bioconductor.org/packages/Biostrings.) We chose the first rotation of the sequence that gave the longest un-gapped alignment anchored to either the start or end of the read. The adapter sequence was then trimmed from the rotated consensus sequences to yield the final trimmed consensus sequences.

### Determination of accuracy

We assessed consensus sequence accuracy using a synthetic 21 nt oligo with the sequence of human miRNA hsa-let7a (S1 Table, oligo 1). The oligo was prepared and sequenced as described above with both the amplification and no amplification workflows. We aligned the consensus sequences obtained to the expected sequence of the oligo using a local alignment with soft-clipping using the smith-waterman algorithm as implemented in the R package Biostrings (R package, version 2.62.0. https://bioconductor.org/packages/Biostrings.) We then determined the edit distance between the consensus sequence and the reference including gaps, insertions, and mismatches. The edit distance was subtracted from the number of aligned bases to determine the percent identity to the reference sequence.

### Read mapping

MiRXplore libraries were mapped to the miRXplore reference using bbmap and read counts were generated during mapping. Human RNA libraries were mapped to the human genome (GRCh38) using bowtie [24] with a seed length of 10 bp and allowing up to 3 mismatches. Reads were counted using htseq-count [25] with the miRNA annotation file from miRbase [26] or the tRNA annotation file downloaded from USCS genome browse [27].

### MiRXplore normalization

Considering the 962 miRNAs in miRXplore were present in equimolar amounts, an expected read count was generated by dividing the total number of mapped reads for each library by 962. Reads were then normalized by dividing the raw read counts by the expected read counts. An miRNA represented with the exact expected read count would have a normalized value of 1; over- and under-represented sequences would have values greater and lower than 1, respectively.

## Results

### Induro RT enables rolling circle reverse transcription

**Fig 1:**
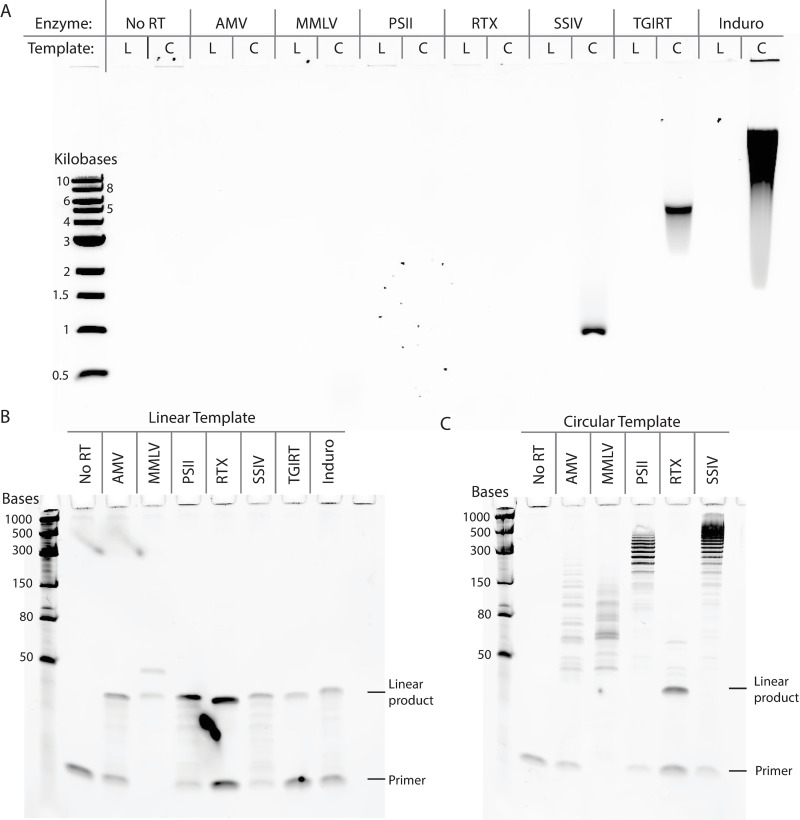
Comparison of rolling circle reverse transcription ability by various reverse transcriptases. (A) Agarose gel electrophoresis was applied to visualize the large cDNA products. Each enzyme was evaluated with both a linear (L) and a circular (C) RNA template. The same reactions products were also resolved by denaturing PAGE gel electrophoresis to visualize the small cDNA products with the (B) a linear RNA template and (C) a circular RNA template.

We compared the ability of various commercially available RTs to perform rolling circle reverse transcription. Five of the tested RTs were derived from retroviruses, including: Avian Myeloblastosis Virus RT (AMV), Moloney Murine Leukemia Virus RT (MMLV), ProtoScript II RT (PSII, an MMLV RT mutant with reduced RNase H activity), SuperScript IV RT (SSIV, an MMLV RT mutant with reduced RNase H activity) and WarmStart RTx (RTx, an *in silico* designed RT with RNase H activity). Two other tested RTs were derived from Group II introns, including thermostable Group II intron RT (TGIRT) and Induro RT. We performed reverse transcription with each RT under the manufacture’s recommended reaction conditions with either a linear or a circularized RNA template. Both linear and circular RNA templates are 42 nt long and share the same sequence. Reaction products were treated with RNase H and RNase If to remove the RNA template and visualized using both agarose gel electrophoresis (for large cDNA products) and denaturing polyacrylamide gel electrophoresis (for short cDNA products). Retroviral RT reactions were allowed to continue for one hour while Group II intron RT reactions were stopped at 15 minutes, because pilot experiments showed that a full hour incubation with the Group II intron RTs would create cDNA products too large to be resolved by the gels. When comparing different RT products resolved by agarose gel electrophoresis, we found that the Group II intron RTs generated larger cDNA products with the circular RNA template, indicating robust rolling circle reverse transcription (Fig 1A). Between TGIRT and Induro RT, we found that Induro RT generated longer cDNA products with a higher overall yield than TGIRT. Among the retroviral based RTs, only SuperScript IV generated enough of the rolling circle product to be visualized on the agarose gel, however the cDNA product was smaller than the ones produced by the Group II intron RTs (Fig 1A). To visualize smaller cDNA products, we ran the other half of each reaction using denaturing PAGE gel electrophoresis (Fig 1B and 1C). With the linear RNA template, we found that all the RTs were able to generate the 42 nt linear cDNA product as expected (Fig 1B). With the circular RNA template, only the retroviral RT reactions were run due to the high molecular weight of cDNA products generated by the Group II intron RT reactions, which were not suitable for PAGE gel electrophoresis (Fig 1C). Among the retroviral RTs, we found that the RT variants with reduced RNase H activity (PSII and SSIV) produced the longest rolling circle cDNA products (300-1000nt). MMLV RT and AMV RT also produced extended products, however their size was limited to under 150 nt, possibly due to intact RNase H activity hydrolyzing the phophodiester backbone of the circular RNA template during reverse transcription. WarmStart RTx produced only the linear product, possibly due to robust RNase H activity, lower strand displacement activity or a combination of both. The short length of the extended products and the ladder like appearance on the gel indicates slow nucleotide incorporation and frequent halting of the retroviral RT, making them unsuitable for robust rolling circle reverse transcription when compared with the Group II intron RTs.

### Small RNA library preparation for nanopore sequencing

**Fig 2:**
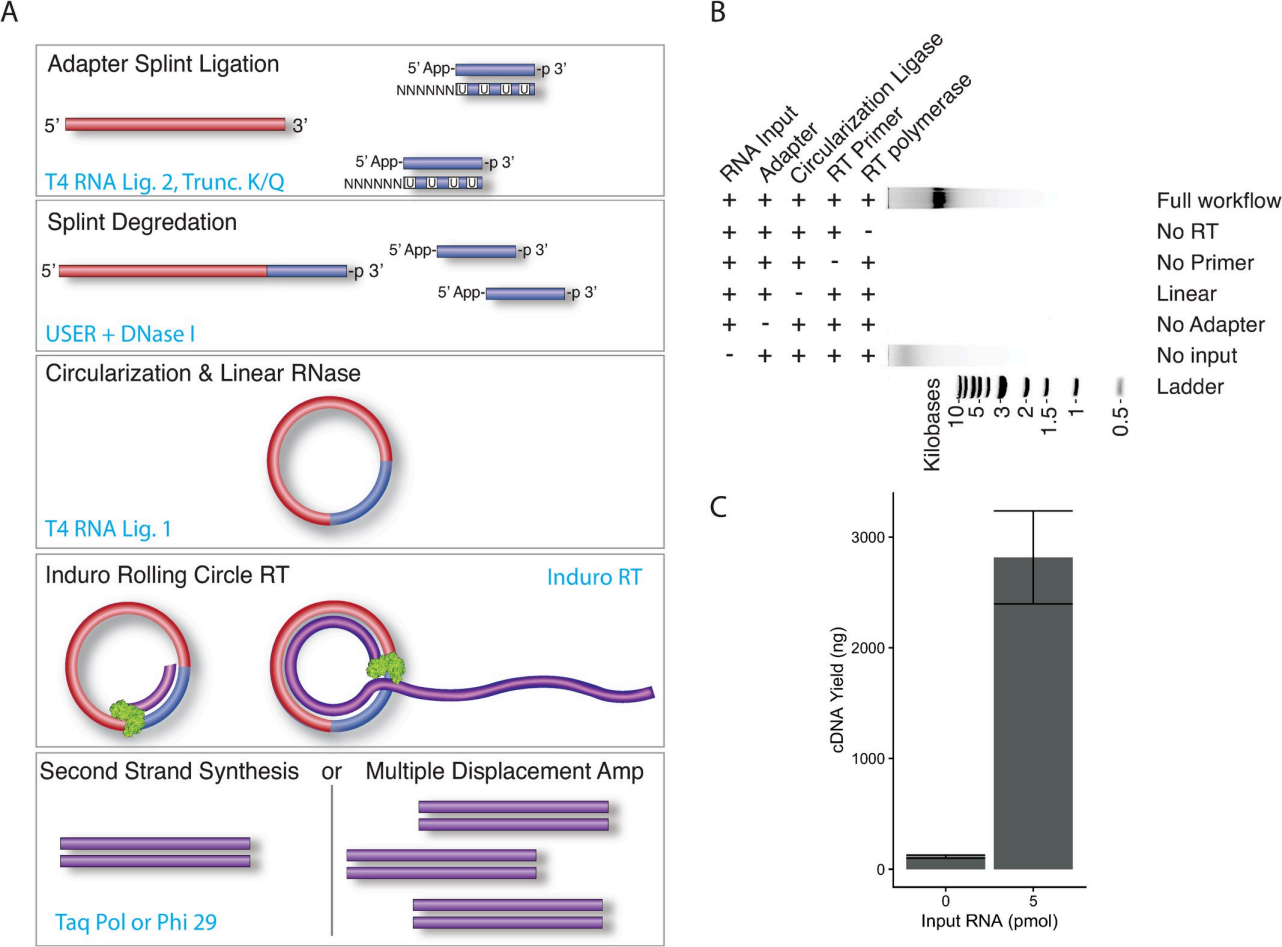
Small RNA library preparation workflow for nanopore sequencing (SR-Cat-Seq). (A) Schematic illustration of SR-Cat-Seq workflow. (B) Component requirement for SR-Cat-Seq. (C) Quantification of the rolling circle reverse transcription reactions with or without RNA input.

Based on the robust rolling circle reverse transcription activity of Induro RT, we reasoned that, by sequencing the long concatemeric cDNA product, it would be possible to enable small RNA sequencing on Oxford Nanopore’s long-read sequencing platform. In addition, the consensus sequence generated by the multiple repeats within the individual cDNA would enhance the accuracy of sequencing. Thus, we created a highly efficient one-pot library preparation workflow to produce concatemeric cDNA from small RNA, called SR-Cat-Seq. Briefly, in the first step an adapter is ligated to the small RNA targets, using the efficient randomized splint ligation strategy (16). Subsequently, the DNA splint strand of the adapter is enzymatically degraded. The ligated small RNAs are then diluted to favor intramolecular ligation and circularized with the addition of T4 RNA ligase, followed by a cocktail of exoribonucleases to degrade any remaining linear RNA. The reactions were then diluted a second time to accommodate the buffer requirement for the next step of rolling circle reverse transcription, which was performed in the same tube with addition of Induro RT (Fig 2A). The resulting concatemeric cDNAs are single-stranded and the second strand synthesis can be performed using Taq DNA polymerase (SR-Cat). Alternatively, if RNA input is low, the single-stranded cDNA can be further amplified using a multiple displacement reaction with phi29 DNA polymerase (SR-Cat-Amp; Fig 2A). We tested the workflow on a library of degenerate 21bp RNA oligos. We found that all components of the reaction (RNA input, the adapter, T4 RNA ligase, primer and Induro RT) were required for robust rolling circle reverse transcription (Fig 2B). In the absence of RNA input we found that a minimal amount of cDNA product was generated (averaging 113 ng ± 13.6), compared to the high yield of product generated from the reaction containing 5 pmol RNA input (averaging 2,816 ng ± 420; Fig 2C). The non-specific product was only seen in reactions that contained the adapter and was therefore probably caused by a small amount of circularized adapter being reverse transcribed by Induro RT in the absence of RNA input.

**Fig 3:**
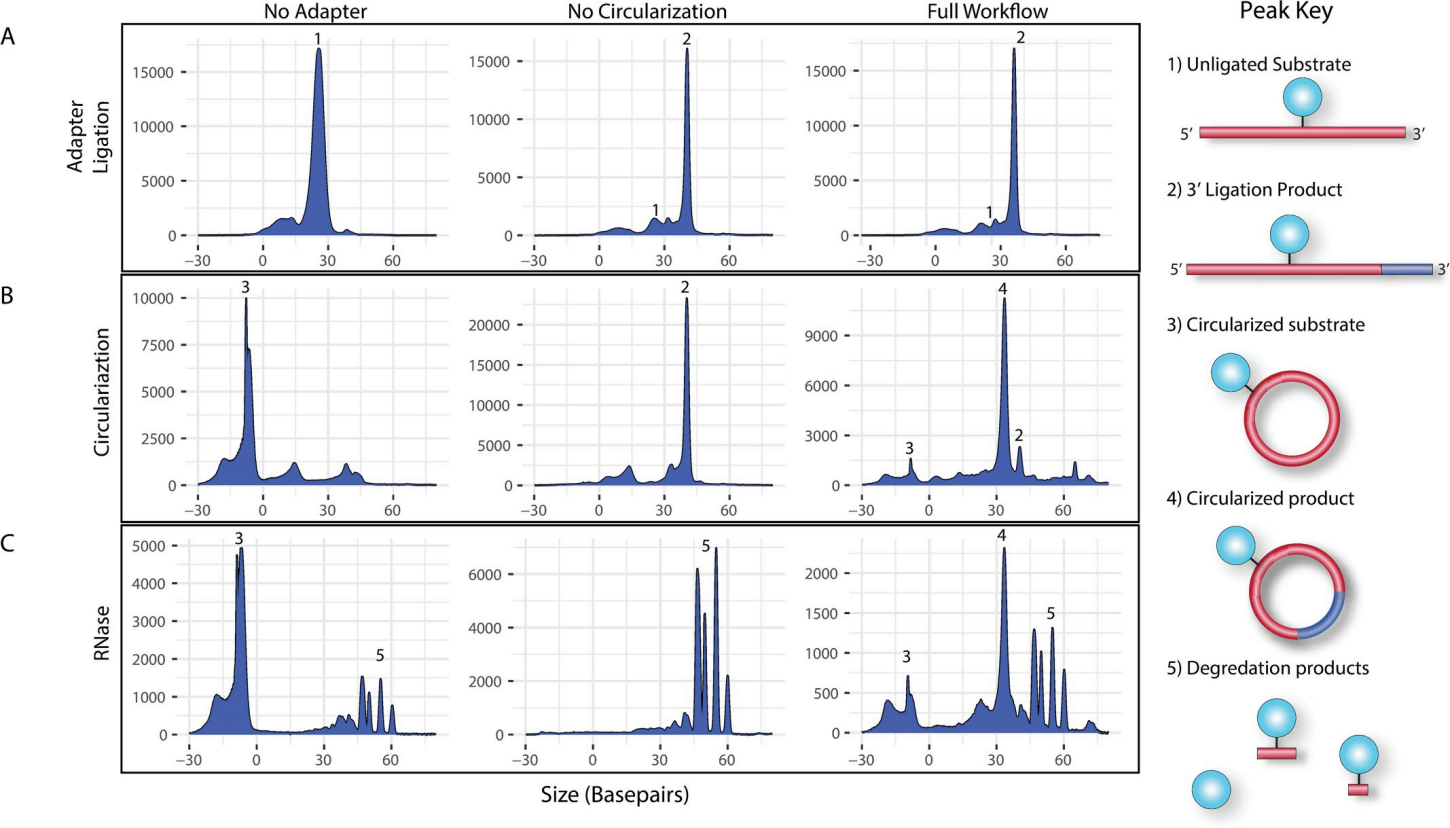
Quantification of small RNA adapter ligation step and circularization step in the SR-Cat-Seq workflow. Three sets of reactions were performed in parallel, with representative capillary electrophoresis traces shown. One set without the adapter (column 1, No Adapter), one set without the ligase (column 2, No Circularization) and one set including both (column 3, Full Workflow). (A) Reactions were sampled after adapter ligation. (B) Reactions were sampled after circularization. Note that the circular products migrate faster through the capillary, making their size appear smaller. (C) Reactions were sampled after exoribonuclease digestion. Note that free FAM dye or very short oligos migrate slower through the capillary due to low charge, which causes them to appear larger than their actual size.

To assess the efficiency of RNA circularization in the SR-Cat-Seq workflow, we used a 21-mer, FAM labeled, degenerate RNA oligo (S1 Table, oligo 8) as the input to mimic the cellular miRNA and quantified the products of each step using capillary electrophoresis (CE). In this experiment we compared 3 reaction conditions: one which did not contain the adapter during the first ligation (Fig 3, column 1, ‘No Adapter’), one which did not contain the T4 RNA ligase during the circularization step (Fig 3, column 2, ‘No Circularization’), and one which had all components (Fig 3, column 3, ‘Full Workflow’). A small aliquot was removed from each reaction after each step of the workflow for quantification using CE. Consistent with our previous study (16), randomized splint ligation was a highly efficient process with 80.8% ± 4.65 of the input RNA converted to the ligated products, compared to 2% ± 0.91 in the negative controls (Fig 3A, column 2 and 3 vs. column 1). Subsequently the reactions were diluted and circularized with T4 RNA ligase 1. Under these reaction conditions we found that the intramolecular ligation (circularization) was highly favored over intermolecular ligation (concatemerization) and the reaction produced 80.5% ± 3.52 circular RNA products and no measurable concatemers, compared to 4.13% ± 8.26 in the negative controls (Fig 3B, column 3 vs. column 2). Finally, to ensure that the circular products were indeed covalently closed circles and identified properly, we treated the reaction products with a mixture of two exoribonucleases (XRN-1 and RNase R). We found that the circular products were, as expected, resistant to the exoribonucleases, while all the linear intermediates were degraded (Fig 3C).

### Nanopore sequencing of concatemeric cDNAs allows for high fidelity reconstruction of the original sequence

**Fig 4:**
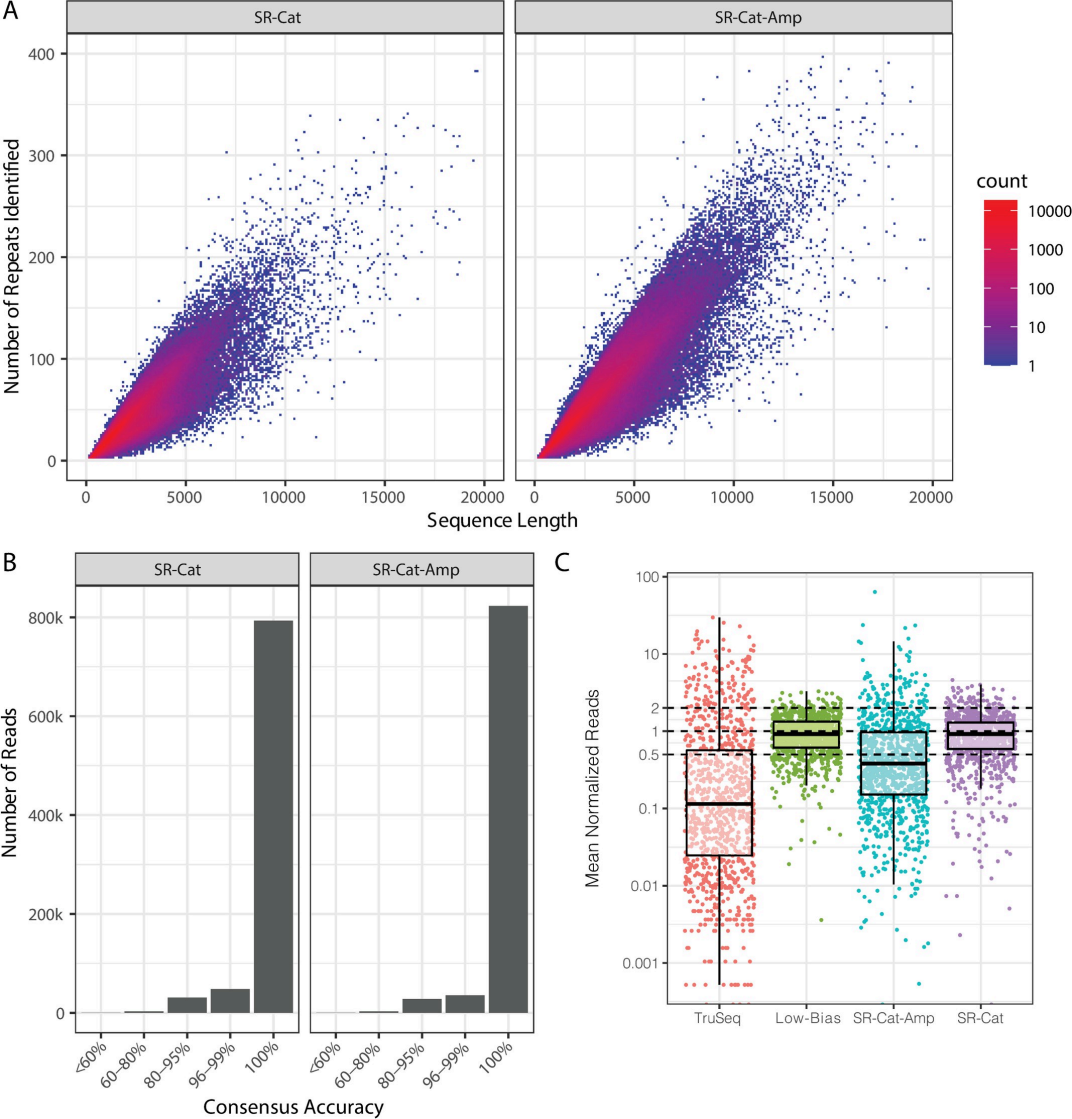
High sequencing accuracy and low bias of SR-Cat-Seq workflow. (A) The number of repeats identified within individual concatemeric cDNA read was plotted against the read length. (B) Consensus sequences assembled from the repeats were mapped to the reference sequence. The accuracy was determined by calculating the percent identity to the reference. The number of reads in each accuracy bin were plotted. (C) Comparison of sequencing bias of four different small RNA sequencing workflows (TruSeq, Low-Bias, SR-Cat-Amp and SR-Cat). Normalized value of each miRNA in miRXplore was plotted for each workflow.

To assess the sequencing accuracy of the SR-Cat-Seq workflow, we sequenced a 42-mer RNA oligo, containing the sequence of a human miRNA (hsa-let7a) on the 5’ end and our adapter sequence on the 3’ end, mimicking the ligation product in the first step of the workflow with a fully known sequence. This oligo was circularized and rolling circle reverse transcription was performed using our optimized workflow. The resulting concatemeric cDNA underwent either second strand synthesis (SR-Cat) or amplification by phi29 DNA polymerase (SR-Cat-Amp). We sequenced the libraries and applied the SPADE algorithm [23] to identify concatemeric repeats in the raw reads and assemble consensus sequences. We found a strong correlation of read length and the number of repeats within the read for both SR-Cat and SR-Cat-Amp, as expected (Fig 4A). The repeats were then aligned to generate a consensus sequence. The adapter was trimmed from the consensus sequence and then the trimmed consensus sequence was aligned to the reference sequence to determine the accuracy. We found that the consensus reads were highly accurate with >96% of reads having at least 95% accuracy and >90% of reads with 100% accuracy (Fig 4B).

To evaluate the sequencing bias of the SR-Cat-Seq workflow, we sequenced a control set of RNA oligos called miRXplore. MiRXplore is a pool of 962 synthetic miRNAs which are mixed at equimolar concentration and commonly used to benchmark bias in small RNA sequencing workflows. To quantify the sequencing bias, we normalized the read counts such that reads represented in the library at their expected values would have a normalized count of 1, while over-represented reads had a normalized count > 1 and under-represented reads had a normalized count < 1 (Fig 4C). The bias was then quantified by calculating the percentage of reads that had a normalized count within 2-fold of the expected value. We compared our data to previously published data generated using short-read Illumina sequencing platform (16), including a standard protocol (TruSeq, S1 Fig) as well as a low bias short read method that uses randomized splint ligation strategy (Low-Bias, S1 Fig). We found that overall, both SR-Cat and SR-Cat-Amp had lower bias than TruSeq, which had an average of only 13.2% of reads within 2-fold of the expected value (Fig 4C). The SR-Cat workflow had 73.1% of reads within 2-fold of their expected value which was comparable to the low-bias short read workflow (78.1%), while the SR-Cat-Amp workflow had only 31.2%, indicating that the multiple displacement amplification step introduces a significant amount of bias (Fig 4C).

### Sequencing human brain small RNA with SR-Cat-Seq workflow

**Fig 5:**
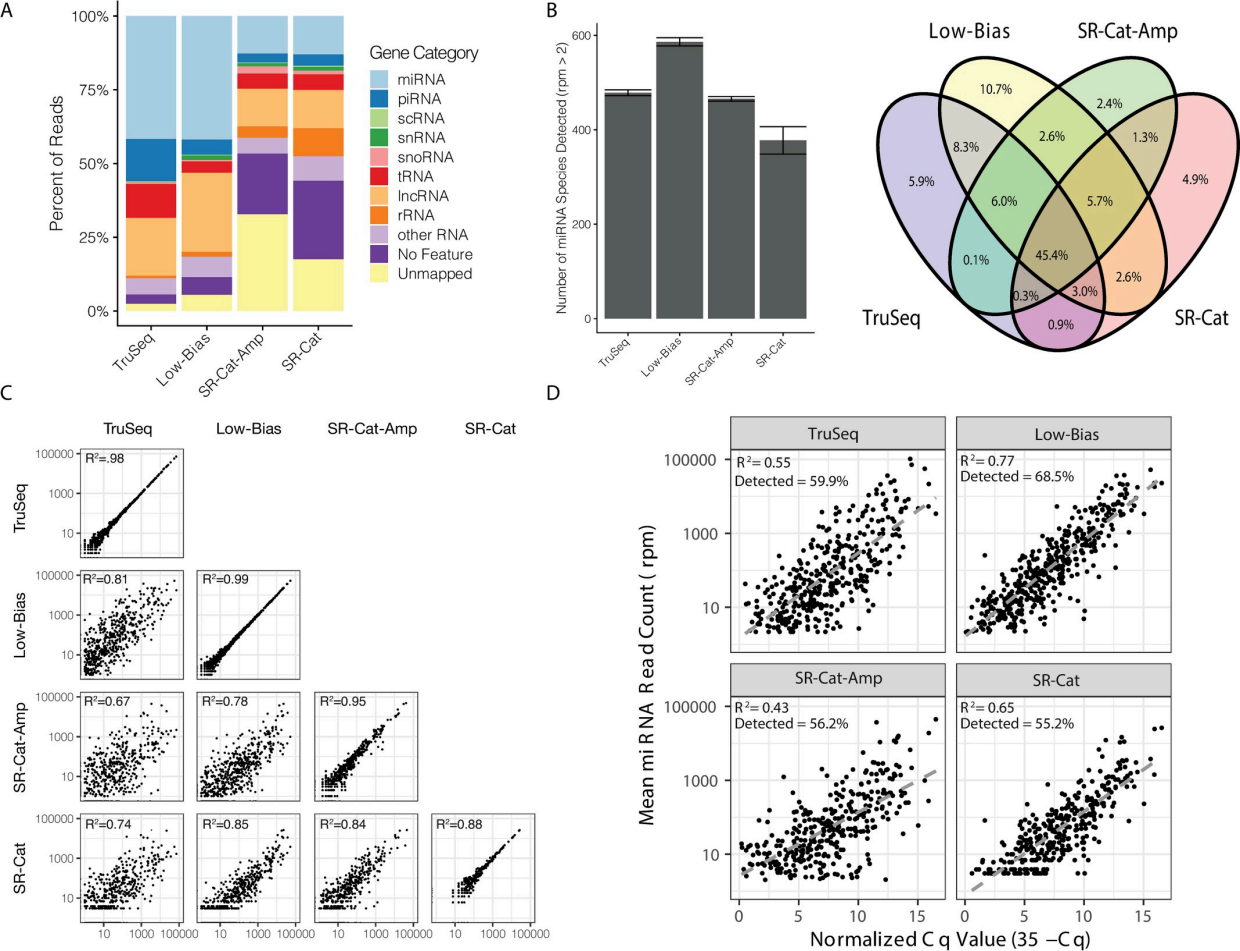
Sequencing human brain small RNA with SR-Cat-Seq workflow. (A) Comparison of the relative abundance of the different ncRNA categories with four different small RNA sequencing workflows (TruSeq, Low-Bias, SR-Cat-Amp and SR-Cat). (B) Left: Comparison of the number of unique miRNA species detected by four workflows. Right: Venn diagram showing overlap of miRNA species detected among the four workflows. C) Correlations of read counts of miRNA in human brain RNA among the four workflows. The diagonal shows the correlation of two technical replicates of each method. R^2^ values are plotted in the upper-left corner of each plot. D) Correlation of miRNA read counts from each workflow compared to qPCR cycle threshold values from human brain RNA. R^2^ values and the percentage of miRNAs detected with rpm > 2 by each sequencing method are plotted in the upper-left corner of each plot.

We then applied the workflow to sequence small RNA in human brain samples. The same brain RNA was sequenced with 2 technical replicates for both the SR-Cat and SR-Cat-Amp workflows. We detected various sRNAs such as miRNA, piRNA and tRNA as well as some long non-coding RNA and ribosomal RNA (Fig 5A). Both SR-Cat and SR-Cat-Amp workflows had similar non-coding RNA content. Compared to the short-read sequencing strategies (TruSeq and Low-Bias), both SR-Cat and SR-Cat-Amp workflows had significantly more percentage of unmapped reads and a lower percentage of miRNA containing reads. We examined the detected miRNAs in more detail. We found that the SR-Cat-Amp had a similar detection sensitivity to standard short-read sequencing (TruSeq) and less detection sensitivity than the Low-Bias workflow. The SR-Cat workflow without amplification had the lowest detection sensitivity (Fig 5B). Comparing the content of all four libraries, we found that 45.4% of the miRNAs were detected by all 4 workflows (Fig 5B). Furthermore, we looked at all the pairwise correlations of miRNA levels between the different workflows as well between technical replicates of each workflow. We found that all the workflows had strong positive correlations between replicates, indicating repeatability of each workflow (Fig 5C, diagonal). We also found significant positive correlations between all the workflows, with some variability introduced by the different workflows (Fig 5C, off-diagonal). To compare to a method independent of biases introduced by sequencing library preparation, we compared each workflow to a previously published qPCR dataset performed on the same RNA sample [16]. We found that the SR-Cat and SR-Cat-Amp workflows detected similar miRNAs as TruSeq, but less than Low-Bias workflow. The Low-Bias method had the strongest positive correlations, and the SR-Cat-Amp had the weakest positive correlations with the qPCR data (Fig 5D).

## Discussion

In this work we show that Group II intron RTs have strong strand displacement activity and are capable of quickly producing large concatemeric cDNAs from circular RNA templates. Based on this activity we developed SR-Cat-Seq, a highly efficient one-pot workflow that enables sRNA sequencing on the nanopore platform. The whole workflow includes adding adapters to sRNAs, circularization and rolling circle reverse transcription to produce concatemeric cDNAs from small RNAs. We showed that SR-Cat (without amplification) workflow has minimal sequencing bias, which is comparable to the Low-Bias workflow. However, the amplification step in the SR-Cat-Amp introduced bias to the whole workflow, which remains to be improved. Workflows with low sequencing bias are important in applications such as liquid biopsy where accurate abundance measurements can help to rank targets and guide real-time treatment decisions. Finally, we applied the workflow to sequence small RNAs from human brain RNA and demonstrated similar accuracy and sensitivity to Illumina’s TruSeq, the standard short-read method. In addition, we developed a bioinformatics workflow that can reconstruct highly accurate consensus sequences from the concatemeric cDNA products. High fidelity sequencing is extremely important for small RNA research. Because of the small size of small RNAs, even single mismatch may make mapping difficult and cause incorrect counts. Furthermore, for liquid biopsy applications, tumor specific isomirs are of great interest and high-fidelity sequencing is required for detection.

We tested versions of our workflow with and without amplification and found that there were advantages and disadvantages to each. The unamplified workflow (SR-Cat) had the lowest bias and would therefore be the best option to use if an accurate representation of the sRNA transcriptome was desired. However, it also had disadvantages including high input requirements of at least 5 pmol for oligonucleotides or 50 ng of sRNA purified from total RNA. These input requirements would preclude the use of this method for most patient samples without further development. Furthermore, the unamplified workflow had a much higher fouling rate of the nanopores which limited the overall output from each flowcell (S2 Fig). While some variability of total output is expected between flow cells, we observed that, in the SR-Cat workflows, the data output began to plateau after less than 24 hours, compared to 40–60 hours for the workflow with amplification (SR-Cat-Amp). We speculate that this was caused by incomplete second strand synthesis and possible aggregation of the concatemeric cDNAs which may have clogged the pores. It is possible that further optimization of the second strand synthesis protocol and/or the use of nuclease flushing followed by reloading of the flowcell could alleviate this problem (Oxford Nanopore EXP-WSH004). In contrast, the amplification workflow (SR-Cat-Amp) utilized phi29 DNA polymerase to amplify the concatemeric cDNAs using multiple displacement amplification. This allowed for less of the sample to be used (only 40 ng of rolling circle cDNA was used compared to 1 ug in the unamplified workflow). Furthermore, this amplification process alleviated the nanopore fouling problem and the lifetime of the flowcell was normal with these samples allowing for higher output per flowcell. However, the MDA (multiple displacement amplification) process introduced significant bias as evidenced by the higher bias with the miRXplore reference sample and low correlation to the unamplified samples. It is possible that a different amplification scheme could be used to realize the benefits of amplification with a better bias profile. Possibilities include PCR, which could be done using the adapter as a priming site and a polymerase that has 5’-3’ exonuclease activity to degrade primers annealed internally to cDNA, or MALBAC which is an isothermal method that has been reported to reduce some of the shortcomings of MDA [28]. On-going work in our laboratory will further develop this process to allow for low-input diagnostic applications.

The ability of Group II Intron RTs to perform rolling circle amplification has many possible applications beyond the one explored in this work. Analogous activity in DNA polymerases, such as phi29 DNA polymerase, has enabled many different technologies such as whole genome amplification [29] and *in situ* sequencing [30] among many others. Furthermore, this activity will be beneficial for the study of a recently discovered class of non-coding RNA, naturally occurring circular RNA, which are of great interest in the fields of RNA biology, cancer biology and RNA therapeutics.

## Supporting information

S1 Fig Schematic illustration of ligation strategies of TruSeq, Low-Bias seq and SR-Cat/SR-Cat-Amp seq. (TIF)

S2 Fig SR-Cat-Amp libraries preserve pore function longer than SR-Cat libraries. Cumulative number of reads sequenced is shown on the y-axis vs time in hours on the x-axis, each line represents an individual MinION flow cell.
(TIF)

S1 Table Oligos used in this study. (DOCX)

S1 Raw images (PDF)
