# Supplementary figures and images for: Rolling circle reverse transcription enables high fidelity nanopore sequencing of small RNA
Source: PLoS One. 2022 Oct 10;17(10):e0275471. doi: 10.1371/journal.pone.0275471 (PMC9550094; doi:10.1371/journal.pone.0275471)

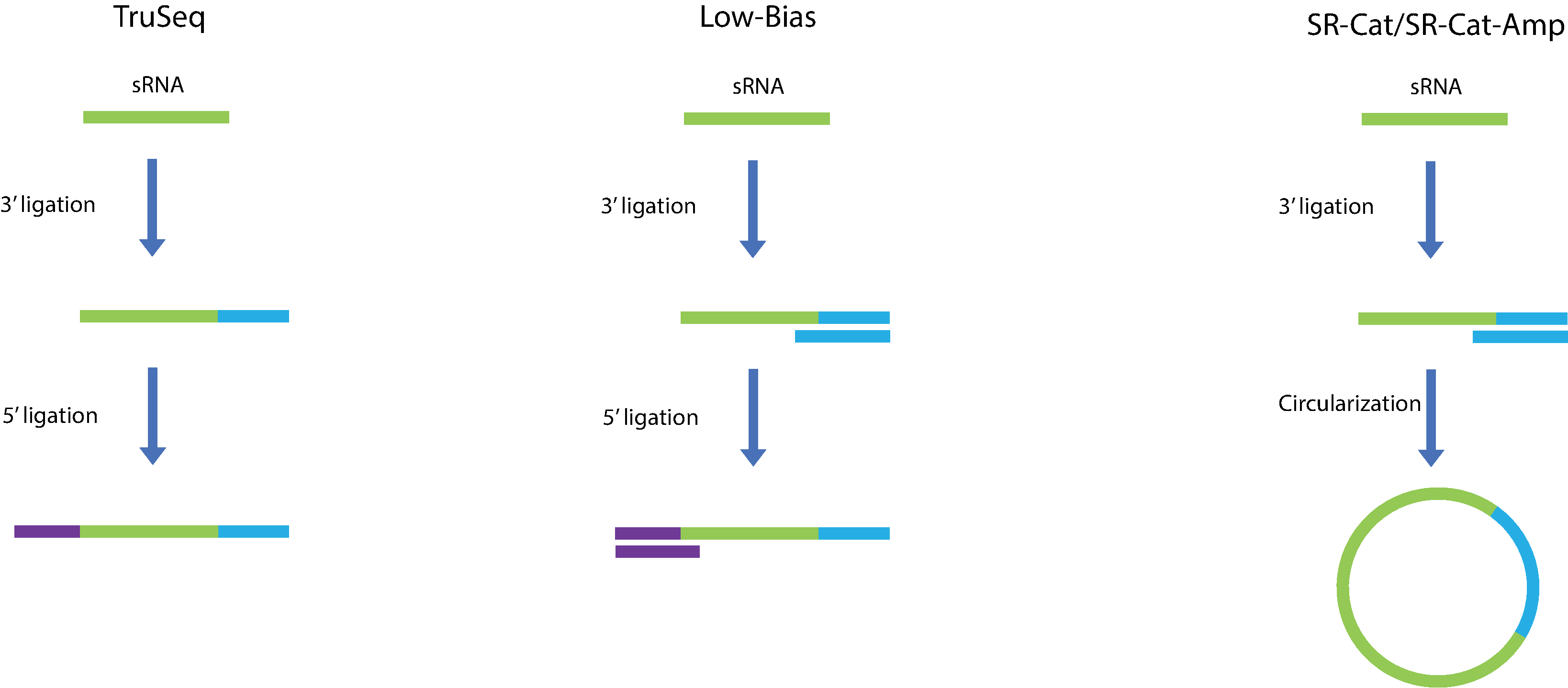

Supplement: S1 Fig — (TIF) [file pone.0275471.s001.tif]

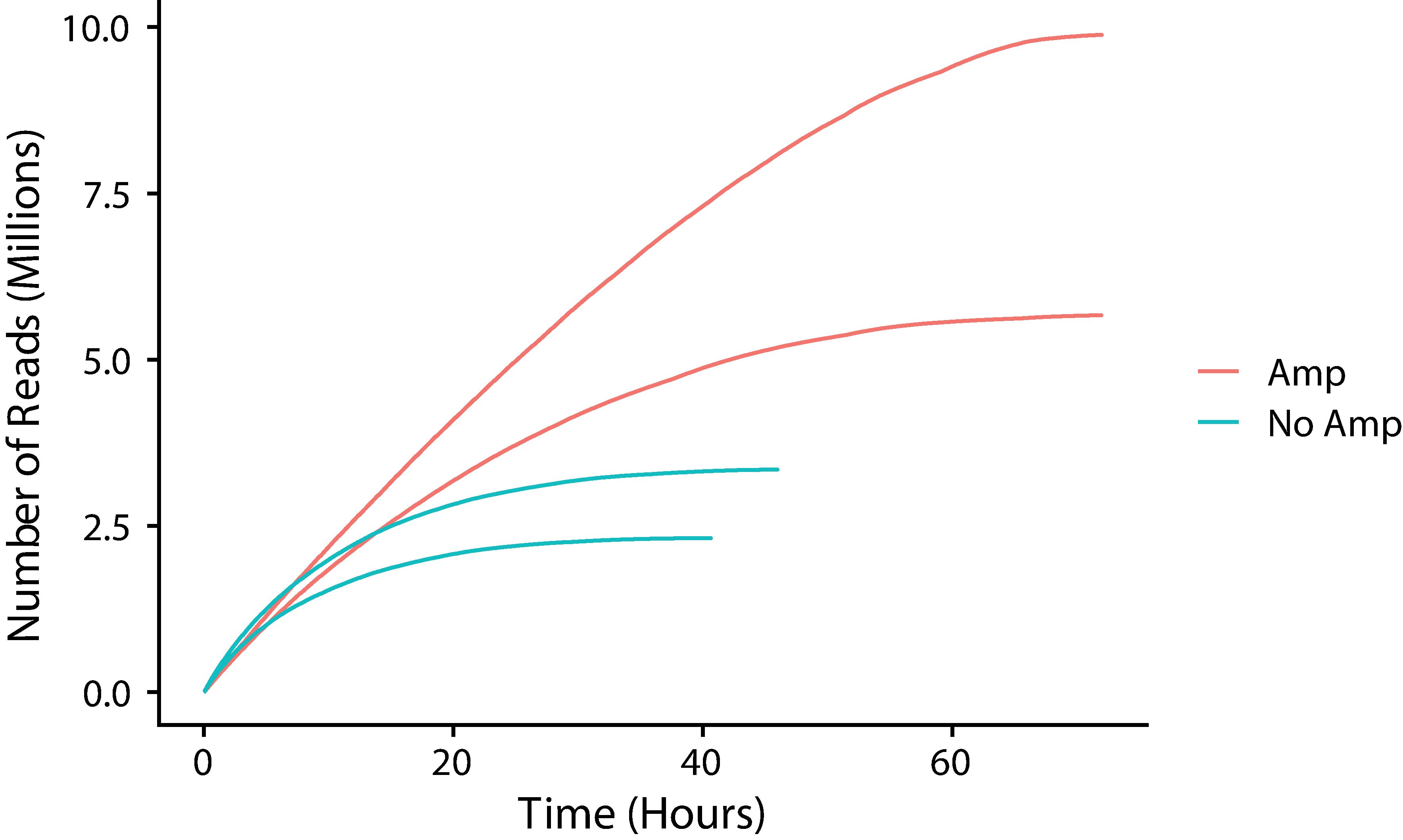

Supplement: S2 Fig — Cumulative number of reads sequenced is shown on the y-axis vs time in hours on the x-axis, each line represents an individual MinION flow cell. (TIF) [file pone.0275471.s002.tif]

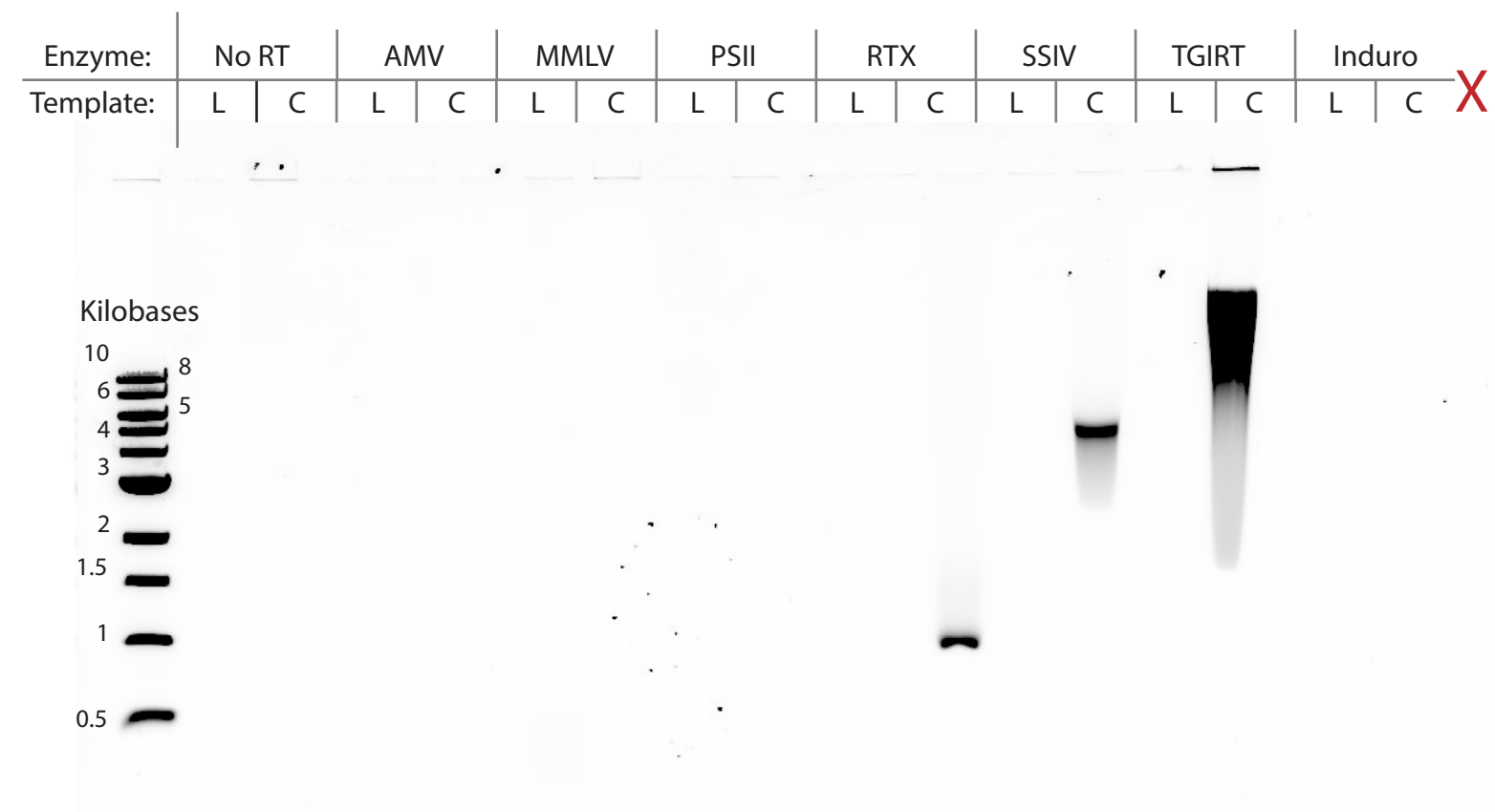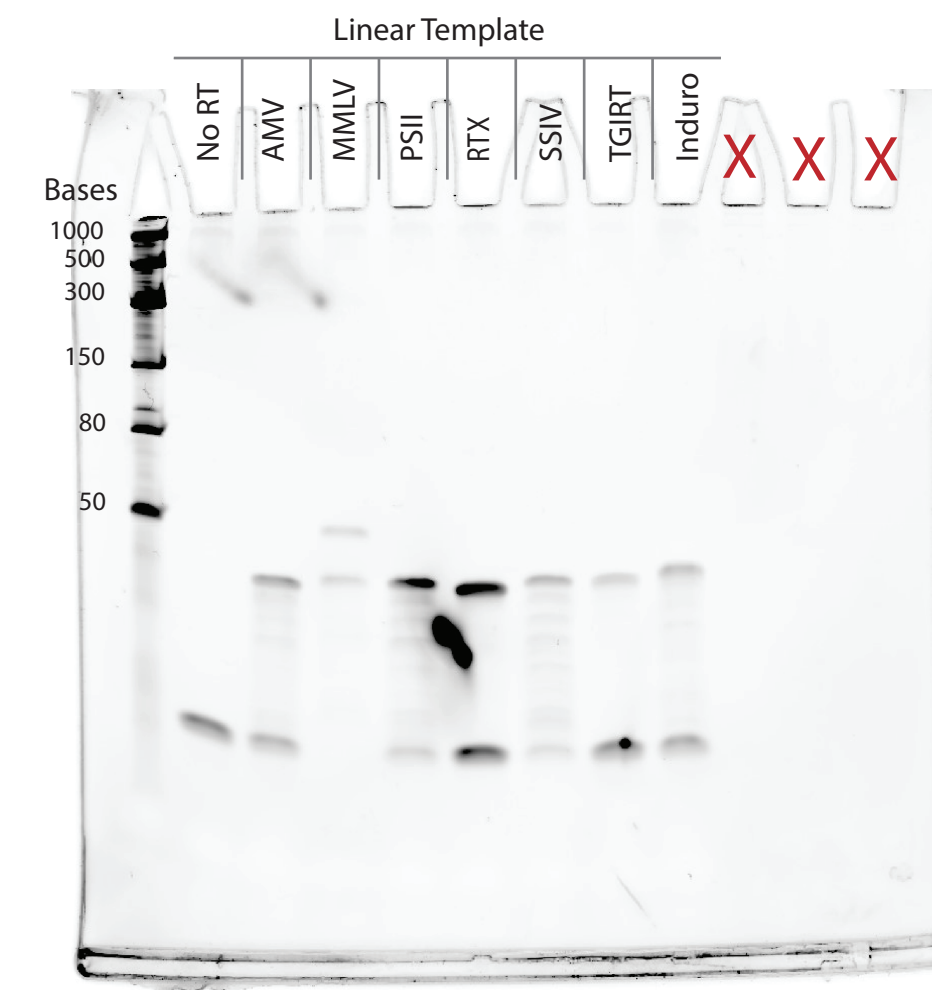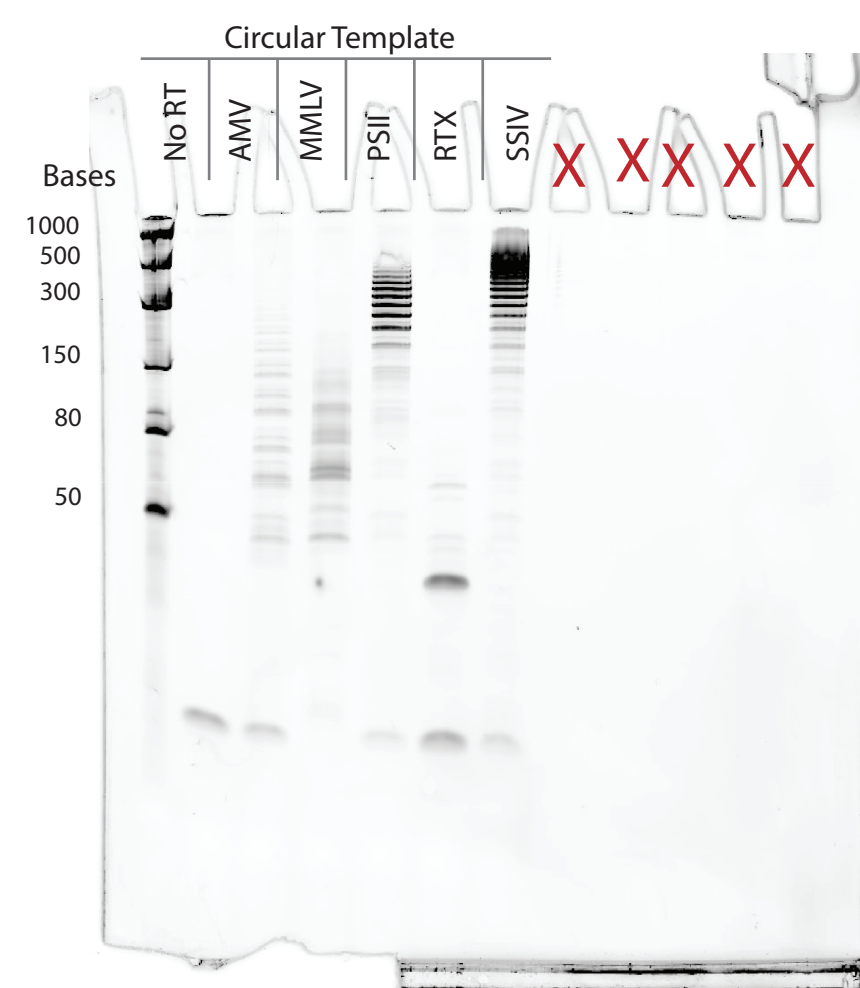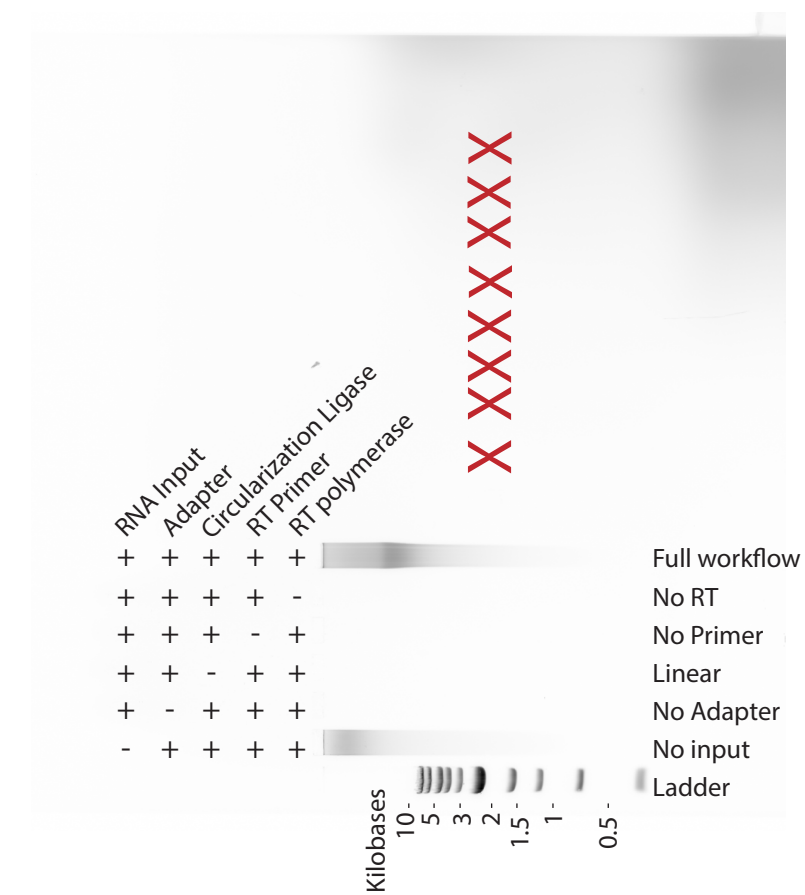

Supplement: S1 Raw images — (PDF) [file pone.0275471.s004.pdf]
