# Supplementary material for: Rolling circle reverse transcription enables high fidelity nanopore sequencing of small RNA
Source: PLoS One. 2022 Oct 10;17(10):e0275471. doi: 10.1371/journal.pone.0275471 (PMC9550094; doi:10.1371/journal.pone.0275471)
Supplement: Supplementary file 2 [file pone.0275471.s003.docx]

Oligo Number	Oligo Name	Sequence
1	HSA-Let7a-Control	/5phos/rUrGrArGrGrUrArGrUrArGrGrUrUrGrUrArUrArGrUrUrUrArCrUrGrArCrCrArGrGrArCrGrArCrGrArCrA
2	RT Primer	TGTCGTCGTCCTGGTCAGTA
3	Top RNA strand	/5Phos/rUrArCrUrGrArCrCrArGrGrArCrGrArCrGrArCrA/3Phos/
4	Bottom DNA splint strand	UGUCGUCGUCCUGGUCAGUANNNNNN/3InvDT/
5	Second Strand Synthesis Primer	TACTGACCAGGACGACGACA
6	MDA_p1	TACTGACC*A*G
7	MDA_p2	TGTCGTCG*T*C
8	rN-21-Internal FAM	/5phos/rNrNrNrNrNrNrNrNrNrN/i6-FAMK/rNrNrNrNrNrNrNrNrNrN

Key:

/5phos/ = 5' Phosphorylation
/3phos/ = 3' Phosphorylation
/3InvDT/ = 3' Inverted dT
* = Phosphorothioate bond
rN = Ribonucleotide
/i6-FAMK/ = FAM dye attached to a dT base
